# Reduced frontal white matter microstructure in healthy older adults with low tactile recognition performance

**DOI:** 10.1038/s41598-021-90995-w

**Published:** 2021-06-03

**Authors:** Focko L. Higgen, Hanna Braaß, Winifried Backhaus, Robert Schulz, Gui Xue, Christian Gerloff

**Affiliations:** 1 Department of Neurology, University Medical Center Hamburg-Eppendorf, Martinistraße 52, 20246 Hamburg, Germany; 2 Key Laboratory of Cognitive Neuroscience and Learning, Beijing Normal University, Beijing, 100875 China

**Keywords:** Neurology, Cognitive ageing, Sensory processing

## Abstract

The aging of the nervous system is a heterogeneous process. It remains a significant challenge to identify relevant markers of pathological and healthy brain aging. A central aspect of aging are decreased sensory acuities, especially because they correlate with the decline in higher cognitive functioning. Sensory and higher cognitive processing relies on information flow between distant brain areas. Aging leads to disintegration of the underlying white matter tracts. While this disintegration is assumed to contribute to higher cognitive decline, data linking structural integrity and sensory function are sparse. The investigation of their interrelation may provide valuable insight into the mechanisms of brain aging. We used a combined behavioral and neuroimaging approach and investigated to what extent changes in microstructural white matter integrity reflect performance declines in tactile pattern recognition with aging. Poor performance in older participants was related to decreased integrity in the anterior corpus callosum. Probabilistic tractography showed that this structure is connected to the prefrontal cortices. Our data point to decreased integrity in the anterior corpus callosum as a marker for advanced brain aging. The correlation between impaired tactile recognition and disintegration in frontal brain networks could provide an explanation why the decrease of sensory function predicts cognitive decline.

## Introduction

Physiological aging has a great impact on the integrity of the peripheral and the central nervous system. This aging of the nervous system is a very heterogeneous process and it remains a significant challenge to identify biologically relevant markers of healthy and pathological brain aging^1,2^.

The age-related alterations affect various cerebral processes from the basic processing of sensory stimuli to higher cognitive functioning such as attention or working memory^3–6^. Sensory impairments with age affect all modalities, e.g. visual acuity and auditory and tactile thresholds, and have a significant impact on the independence and the activities of daily living^4,7–9^. The processes causal for the deterioration of sensory processing seem to be of critical relevance in the course of the aging of the nervous system, as the decline in sensory acuities has repeatedly been shown to correlate with the decline in higher cognitive functioning with age^10–14^. Therefore, the investigation of sensory processing might give valuable insight into the mechanisms of brain aging.

Two main hypotheses are being discussed to explain the relationship between the decline in sensory processes and cognitive domains, the ‘cascade hypothesis’ and the ‘common cause hypothesis’^11,15,16^.

The ‘cascade hypothesis’ proposes that the degeneration of sensory organs leads to sensory deficits and eventually to cognitive decline. In the tactile domain for example, aging leads to morphological changes of mechanoreceptors as well as a decrease in number, resulting in a decline in cutaneous sensation^17,18^. Furthermore, a loss of distal large myelinated sensory fibers and a decrease in thickness of the myelin lead to a decline in sensory nerve conduction^18^. In the view of the ‘cascade hypothesis’, the impairment of the somatosensory system arising from these alterations leads to sensory deprivation and ultimately to a decline in higher cognitive functions.

Of note, earlier studies indicated that in younger adults sensory impairments have rather small effects on higher cognitive domains^19,20^. Furthermore, artificial reduction of sensory acuity does not lower cognitive performance in younger adults^21^. These data argue against the cascade view and indicate that the relevant mechanisms of brain aging unfold their effects on higher stages of the sensory processing stream.

Central sensory processing relies on local brain activation and interregional information flow. Especially the processing of complex stimuli requires an interplay between primary and higher-order sensory areas^22,23^.

For tactile recognition, there is an interaction of bottom-up sensory flow with top-down control^24–26^. Bottom-up tactile inputs are primarily processed in the primary somatosensory cortex (S1) and then segregated into different pathways for different object properties. Relevant cortical areas of this distributed network include the parietal operculum (SII), the posterior parietal cortices, the intraparietal sulcus, the temporo-parietal junction and the limbic areas^25,27,28^. Accordingly, structural and functional integrity in primary or secondary sensory areas have been linked to sensory capabilities with age. For example, an age-related decrease in grey matter integrity has been shown in postcentral gyrus^29^ as well as changes in primary sensory cortex BOLD response^30^. Another common finding is a less-lateralized pattern of activity across the primary sensory cortices^31^.

Concurrently, tactile processing is mediated by higher cognitive functioning such as top-down attentional control and visuo-spatial working memory. These functions are executed via inputs from the prefrontal cortices (PFC), comprising, for example, the dorsolateral prefrontal cortex (DFPLC) and the ventrolateral prefrontal cortex (VLPFC)^24,25,32,33^.

Information flow between the different brain regions requires proper microstructural integrity of the underlying white matter tracts. Structural alterations in both networks of bottom-up sensory flow and networks of top-down modulation, could lead to disturbances in sensory processing. In line with this, the ‘common cause hypothesis’ proposes that the relationship between sensory and cognitive deficits is mediated by a common neuroanatomical basis, e.g. a widespread neural degeneration. In this case, sensory acuities would be indicators of the physiological integrity of the aging brain. While structural disintegrity in bottom-up sensory networks could explain the decline in sensory processing with age, alterations in networks of top-down modulation could constitute an explanation for the correlation of sensory and cognitive measures.

Multiple neuroimaging studies related structural brain integrity to processes of aging. A measurement widely accepted to describe brain network integrity is fractional anisotropy (FA), a parameter derived from diffusion-weighted brain imaging. Within the limitations of fiber-tracking, FA is commonly referred to as a neuroimaging index of microstructural white matter integrity^34–37^. In the following, we will use the term accordingly.

A common finding is a widespread reduction of regional FA with increasing age^38–45^. This so-called ‘cortical disconnection’ is thought to contribute to age-related cognitive decline^46^. The domains affected by the decrease of microstructural white matter integrity are primarily involved in higher cognitive functioning such as executive functioning, processing speed, and memory (for review see^45^). So far, data linking these age-related structural alterations with sensory processing are sparse^47,48^.

Aiming to determine group differences in sensory processing between older and younger participants, we implemented a tactile pattern recognition task. All participants underwent structural brain imaging, including diffusion weighted imaging to characterize global white matter microstructure.

We hypothesized that differences in microstructural white matter integrity as a consequence of brain aging are related to variable success in sensory processing at the behavioral level. Besides the investigation of mechanisms relevant for sensory processing, this approach might also shed light on the link between sensory and cognitive deficits with age.

## Methods

### Participants

37 older and 22 younger volunteers were screened for the study. 6 older volunteers did not meet the inclusion criteria during the initial assessment. 2 older and 2 younger participants dropped out because of personal or technical problems. During task performance, 10 older participants did not meet the predefined accuracy targets (as described below). Older participants were regrouped into O-LP (older-low-performers) and O-HP (older-high-performers). Thus, 10 O-LP (5 female, mean age 74.1, range 68–82), 19 O-HP (11 female, mean age 71.9, range 65–79) and 20 younger participants (= Young, Y; 11 female, mean age 24.1, range 20–28) entered in the final analyses. The group of younger participants as well as O-HP also participated in an already published consecutive study focusing on behavioral performance in visuo-tactile crossmodal pattern matching^6^.

All participants were right-handed according to the Edinburgh handedness inventory^49^, had normal or corrected to normal vision, no history or symptoms of neuropsychiatric disorders (MMSE ≥ 28, DemTect ≥ 13) and no recent history of centrally acting drug intake. All participants received monetary compensation.

### Ethics statement

The study was conducted following the Declaration of Helsinki and approved by the local ethics committee of the Medical Association of Hamburg (PV5085). All participants gave written informed consent.

### Assessment

Before inclusion, each participant underwent an assessment procedure. Latter consisted of a neurological examination, the Mini-Mental State^50^ (MMSE, cut-off ≥ 28) and the DemTect^51^ (cut-off ≥ 13) to rule out symptoms of neuropsychiatric disorders. Furthermore, a 2-point-discrimination test^52^ (cut-off > 3 mm) and a test of the mechanical detection threshold^53^ (MDT, v. Frey Filaments, OptiHair2-Set, Marstock Nervtest, Germany, cut-off > 0.75mN) were conducted to ensure intact peripheral somatosensation. We also assessed subjectively experienced attention deficits with a standardized questionnaire (FEDA). The FEDA consists of the sub-sections A, B and C, where A examines distractibility and slowing of mental processes, B characterizes fatigue and slowing of practical activities, and C focuses on the reduction of energy^54^.

### Task design

The experiment took place in a light attenuated chamber. We chose the experimental procedure, stimulus configuration, and stimulation parameters based on pilot data showing the accuracy of tactile pattern recognition to be very different between older and younger participants.

**Figure 1:**
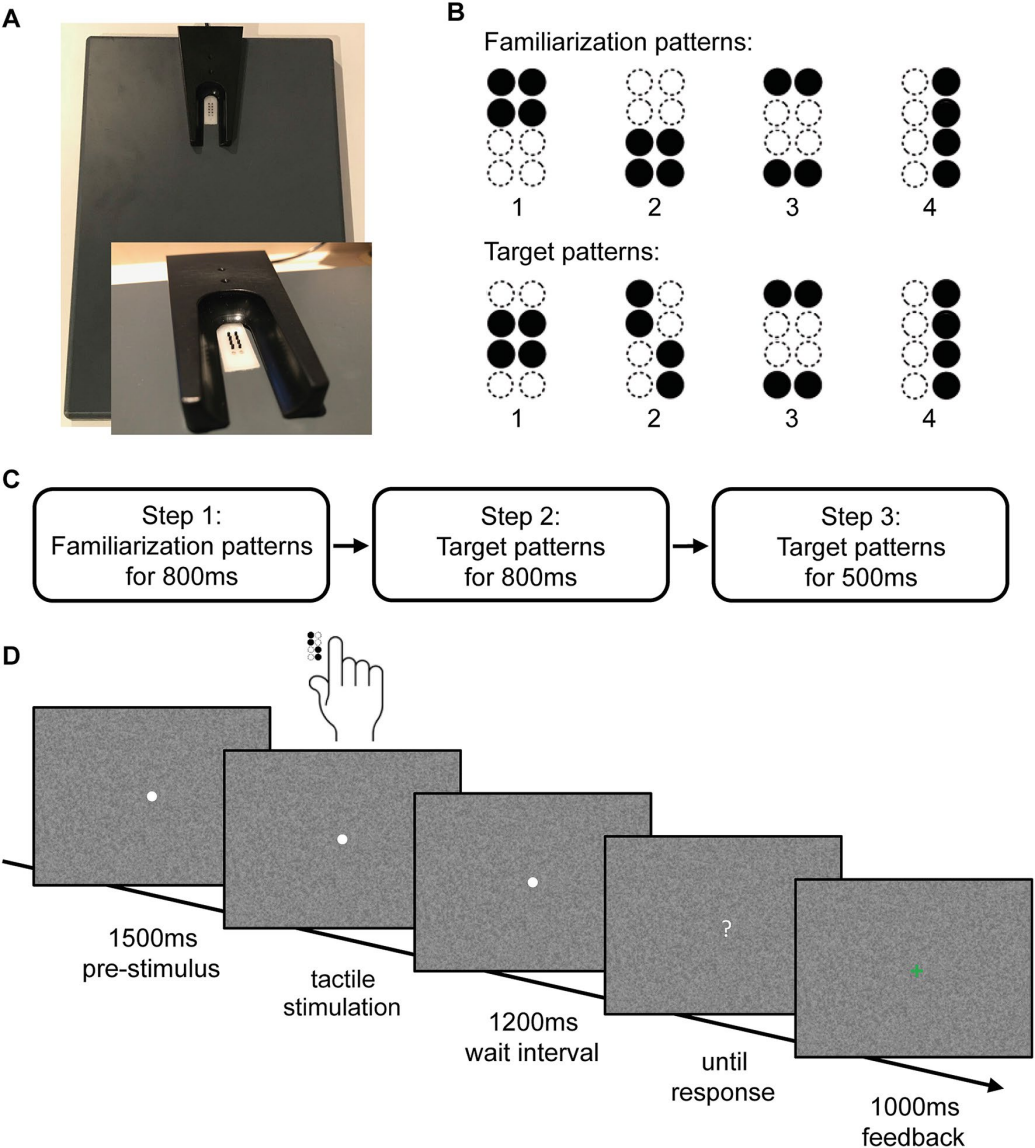
Stimulus design and experimental procedure. (**A**) Braille stimulator. For tactile stimulation, the participants’ right hand was resting on a custom-made board containing a Braille stimulator (QuaeroSys Medical Devices, Schotten, Germany), with the fingertip of the right index finger placed above the stimulating unit. The Braille stimulator consists of eight pins arranged in a four-by-two matrix, each 1 mm in diameter with a spacing of 2.5 mm. Each pin is independently controllable. (**B**) Stimuli consisted of two sets of four tactile patterns, (**C**) Sequence of tasks in the experiment, (**D**) The trial sequence. After a pre-stimulus interval of 1500 ms, tactile patterns were presented to the right index finger with a duration depending on the current step of the experiment. After a wait interval of 1200 ms, a question mark appeared on the screen and participants gave the response via button press. A visual feedback (1000 ms) was provided after every response.

For tactile stimulation, the participants’ right hand was resting on a custom-made board containing a Braille stimulator (QuaeroSys Medical Devices, Schotten, Germany), with the fingertip of the right index finger placed above the stimulating unit (see Fig. 1A). The Braille stimulator consists of eight pins arranged in a four-by-two matrix, each 1 mm in diameter with a spacing of 2.5 mm. Each pin is controllable independently. Pins can be elevated (maximum amplitude 1.5 mm) for any period to form different patterns. At the end of each pattern presentation, all pins return to baseline.

The tactile recognition task represented a delayed match-to-sample task with steps of increasing complexity. After a short familiarization period, participants performed the delayed match-to-sample task over the whole experiment. The different steps only differed in stimulus patterns and trial timing but not in task demands. There was no prior learning of the patterns involved. At the beginning of each step, participants read the task instructions on a computer screen positioned in front of them. The stimuli consisted of different sets of four geometric patterns, each of them formed by four dots (Fig. 1B). The experiment began with a simple set of four familiarization patterns (step 1, Fig. 1B,C) at maximum pin amplitude and a stimulation time of 800 ms to get the participants acquainted with the tactile stimulation.

Each trial started with a central white fixation point appearing on a noisy background, both remained visible throughout every single trial. The tactile pattern presentation started 1500 ms after the appearance of the fixation point with a stimulus chosen pseudo-randomly from the stimulus set. After the tactile presentation, there was a waiting interval of 1200 ms. Then, the central fixation point turned into a question mark and all four possible patterns were presented visually in numbered order on the screen, allowing the participants to indicate which of the four patterns had been presented. Participants responded via button press with the fingers two to five of the left hand. After each trial participants received visual feedback (1000 ms) whether the response was correct (green ‘+’) or incorrect (red ‘−’) (Fig. 1D).

After a minimum of five familiarization blocks, each one consisting of 16 trials, and an accuracy of at least 75% in three of five consecutive blocks, participants could proceed to the next step. If participants did not reach the target accuracy within 15 blocks, they were excluded from further participation.

In the next step of the recognition task, the stimulus set consisted of the four target patterns (step 2, Fig. 1B,C). To train participants in the recognition of the target patterns, stimulation occurred at the maximum amplitude and again with a long stimulation time of 800 ms. Trial timing, blocks and accuracy targets remained as described above. If again, participants were able to recognize patterns with the previously defined accuracy, the stimulation time of the target patterns was decreased to 500 ms as a final step of the recognition task (step 3, Fig. 1B,C). Participants who were able to recognize these patterns with the targeted accuracy were categorized as ‘high-performers’ (O-HP). Participants not reaching this level were labelled as ‘low-performers’ (O-LP). We grouped all participants not reaching the predefined accuracy target at one of the steps of the tactile recognition task together, as they all did not show any deficits in the initial assessment, however a clear performance difference in tactile recognition compared to the younger participants and the older ‘high-performers’. In all these participants alterations in microstructural white matter integrity might be the reason for poor performance, according to our hypotheses.

### Brain imaging

A 3 Tesla MRI system (Magnetom Skyra, Siemens Healthcare, Erlangen, Germany) with a 32-channel head coil acquired diffusion-weighted and high-resolution T1-weighted structural images. For the diffusion-weighted sequence, a spin-echo, echo-planar imaging (EPI) sequence was applied with the following parameters: TE = 82 ms, TR = 10000 ms, flip angle = 90°,matrix size = 104 × 128 matrices, FOV = 208 × 256 mm^2^, voxel resolution = 2.0 × 2.0 × 2.0 mm^3^, partial Fourier factor = 0.75, 75 contiguous transversal slices, one image with b = 0 s/mm^2^, 64 images with b = 1500 s/mm^2^ (64 non-collinear directions). For the T1-weighted sequence, a 3-dimensional magnetization-prepared rapid gradient echo (3D-MPRAGE) sequence was used with the following parameters: TR = 2500 ms, TE = 2.12 ms, TI = 1100 ms, flip angle 9°, 256 coronal slices with a voxel size of 0.83 × 0.94 × 0.83 mm^3^, FOV = 240 mm.

### Image processing

The processing and analysis of MRI data were carried out using FMRIB Software Library (FSL) software 5.0.2.2 (Analysis Group, FMRIB, Oxford, UK Oxford Centre for Functional Magnetic Resonance Imaging of the Brain Software Library, https://fsl.fmrib.ox.ac.uk/fsl/fslwiki/FSL)^55^. First, the eddy current distortion and simple head motion of raw diffusion data were corrected, using Eddy current correction from the FMRIB’s Diffusion Toolbox (FDT) 3.0 (mean displacement: Table S1a and b). Then, the Brain Extraction Tool (BET) v2.1 of FSL was used for brain extraction^56^. FA images were created by fitting a tensor model in each voxel to the raw diffusion data using FDT. Additionally, the eigenvalue images for L1, L2 and L3 were created in the same way and axial (AD) and radial diffusivity (RD) were calculated from the eigenvalues as: AD = L1, RD = (L2 + L3)/2.

### Tract-based spatial statistics (TBSS)

Voxelwise statistical analysis of the created FA data was carried out using TBSS (Tract-Based Spatial Statistics)^57^. All participants’ FA data were nonlinearly registered to the FMRIB58-FA standard-space template (FMRIB Centre University of Oxford, Department of Clinical Neurology, John Radcliffe Hospital Headington, Oxford, UK; https://fsl.fmrib.ox.ac.uk/fsl/fslwiki/FMRIB58_FA) and aligned to the Montreal Neurological Institute (MNI) space using the nonlinear registration tool FNIRT as part of TBSS, which uses a b-spline representation of the registration warp field^58^. Next, the mean FA image was created and thinned to create a mean FA skeleton, which represents the centers of all tracts common to the group. Each participant’s aligned FA data were then projected onto this skeleton and the resulting data fed into voxelwise cross-participant statistics. The permutation-based non-parametric inferences within the framework of the general linear model (GLM, with “GROUP” as factor) were performed to investigate the differences between the groups Y versus O-HP, Y versus O-LP and O-HP versus O-LP using ‘randomise’ (https://fsl.fmrib.ox.ac.uk/fsl/fslwiki/Randomise)^59^. For corrected results, the threshold-free cluster enhancement with the family-wise error (FWE) correction for multiple comparisons corrections (P < 0.05, FWE corrected, 5000 permutations) was used.

The brain region in which the analysis showed significantly different FA-values between the two groups was thresholded at a P-value < 0.05. The result was a region in the anterior corpus callosum (Volume: 2286 voxel (MNI-space, voxel-size = 1 × 1 × 1 mm^3^), center-of-gravity: x = 83, y = 150, z = 84 in MNI-space). This region was first binarized and then used as mask for further analysis. In the next step this mask was back-transformed to the individual diffusion space to create individual masks for each participant. The individual binary masks were then multiplied with the individual FA-maps. We calculated the mean FA in the resulting image for each participant and used the mean FA-values for further statistical calculations.

The same whole brain analysis was performed for AD and RD to investigate the differences between the groups O-HP and O-LP. Additionally, to further explore the underlying microstructural alterations in the seed region in the anterior corpus callosum, AD and RD in this region were computed as described above.

### Probabilistic tractography

After preprocessing the DWI-data with eddycorrect and BET as described above, FSL’s bedpostx was used to estimate the distribution of diffusion parameters in each voxel, modelling crossing fibers using Markov Chain Monte Carlo sampling^60^ (number of fibres per voxel = 3). Probabilistic tractography was used to reconstruct the tracts with the individual masks defined in the anterior corpus callosum (as mentioned above) as seed mask (5000 streamlines sent from each voxel in the individual seed masks, curvature threshold 0.2, steplength 0.5 mm) in each participant. In the next step the individual output distributions were thresholded by 0.01%, 0.5%, 1.0% and 2%, of successful streamlines as described elsewhere^37^. After thresholding, the remaining individual tracts were binarized, transformed into MNI-space and merged for each group. For each threshold, the merged tracts of all participants were plotted and thresholded by 50% [50% lower threshold, in which at least 50% of the participants showed a tract/connection (depending on the respective previous threshold, e.g. 0.1% or 2%)] of all participants of each group (O-HP and O-LP)^37^. We used “randomise” for a statistical comparison of these merged tracts between the two groups O-LP and O-HP^59^.

### Further statistical analyses

Further statistical analyzes were performed using Matlab version 9.1 (R2016b, MathWorks, Natick, MA) and R statistical package Version 3.5.4 (http://www.r-project.org/).

To test for assessment related group-differences a linear model was defined utilizing R’s lm command to investigate the relationship between the assessment variables Age, MDT, 2-point-discrimination, MMSE, DemTect, FEDA-A, FEDA-B, FEDA-C as dependent variables and GROUP (Young, O-HP, O-LP) as independent variable. Age was included into the model to test for age differences in the groups O-HP and O-LP. The comparison was performed using lsmeans (R-package: lsmeans) and pairwise comparison between the resulting contrasts. Benjamini-Yekutieli (BY) was used to adjust for multiple comparisons^61^. For post-hoc testing a MANOVA was used with GROUP as an independent variable and BY correction to adjust for multiple comparisons. Task performance of groups Young and O-HP was compared at each step of the recognition task with a two-sided t-test and BY correction for multiple comparison.

Mean FA, AD and RD-values in the anterior corpus callosum were compared between the groups O-HP and O-LP using a two-sided unpaired t-test. Furthermore, linear regression models were fitted to test for relationships between diffusion parameters (FA, AD and RD) and assessment parameters. Group differences were calculated by means of (diffusion parameter)*GROUP. FDR-correction was performed to correct for multiple comparisons.

## Results

### Behavior

At all steps of the tactile recognition task, younger participants (= Young, Y) performed better than older participants (p < 0.001 at all steps). Besides this expected performance difference between younger and older participants, there were also differences in the accuracy of tactile pattern recognition within the older group. In the older group, 19 of 29 participants were able to reach the predefined accuracy level at all steps. On each step of the tactile recognition task, some older participants failed to reach the predefined target. Five older participants were not able to detect the familiarization patterns with the targeted accuracy. Three older participants failed to detect the target patterns at a stimulation time of 800 ms. Two more older participants failed to detect the target patterns at a stimulation time of 500 ms. These participants were then excluded from further steps. Older participants were regrouped according to their performance in O-HP and O-LP. Taking only the O-HP, the younger participants still performed significantly better at each step (see Table 1, p < 0.001 at all steps). Plots of the accuracies of the three groups at each step of the tactile recognition task are provided in the Supp. Material in Fig. S1 as additional information.

**Table 1:** Performance of the different groups.

Task	Young (n = 20)	O-HP (n = 19)	O-LP (n = 10)
Familiarization patterns for 800 ms	96.9 (± 3.7)*	85.2 (± 9.7)*	47.8 (± 8.0) (n = 5)
Target patterns for 800 ms	93.1 (± 5.2)*	76.4 (± 8.9)*	54.4 (± 1.7) (n = 3)
Target patterns for 500 ms	97.7 (± 5.1)*	82.6 (± 10.7)*	54.4 (± 6.8) (n = 2)

Mean accuracy values over all blocks needed per participant are shown in % ± standard deviation for each step of the tactile recognition task. Group comparisons between Young and O-HP were calculated at each step of the recognition task with a two-sided t-test and BY correction for multiple comparisons.

*Indicate significant differences between Young and O-HP, all p-values ≤ 0.001.

### Assessment

As the factor age was included into the model, group comparison of the data obtained in the assessment prior to inclusion (see Table 2) naturally showed significant differences between Young and O-HP (t(46) = 37.8, p < 0.001) and Young and O-LP (t(46) = 32.2, p < 0.001). Importantly, there was no difference between O-HP and O-LP (t(46) = − 0.93, p = 0.6550).

**Table 2:** Assessment data of the groups.

Metrics	Young (n = 20)	O-HP (n = 19)	O-LP (n = 10)
Age	24.1 (± 2.6)*^+^	71.9 (± 4.4)*	74.1 (± 3.9)^+^
Education (years)	12.5 (± 0.6)	10.7 (± 1.6)	11.7 (± 2.0)
DemTect	17.8 (± 0.6)*	16.0 (± 1.6)*	16.9 (± 1.6)
MMSE	29.7 (± 0.6)	29.5 (± 0.6)	29.2 (± 0.8)
2-Point (mm)	2.1 (± 0.2)	2.2 (± 0.4)	2.4 (± 0.5)
MDT (mN)	0.28 (± 0.1)^+^	0.56 (± 0.5)	0.65 (± 0.4)^+^
FEDA A	4.28 (± 0.4)	4.35 (± 0.4)	4 (± 0.5)
FEDA B	4.55 (± 0.4)	4.65 (± 0.4)	3.97 (± 0.8)
FEDA C	4.35 (± 0.6)	4.38 (± 0.5)	3.75 (± 0.9)

Mean values are shown ± standard deviation. Based on significant main effects, post-hoc tests were conducted.

*Indicate significant differences between Young and O-HP.

^+^Indicate significant differences between Young and O-LP, all p-values ≤ 0.01.

Despite their differences in performance in the tactile recognition task, there were no significant differences between O-HP and O-LP in the assessment data. Post-hoc comparison of the assessment data of Young and O-HP, showed that besides age (F(1, 37) = 1768.1, p < 0.001), DemTect scores (F(1, 37) = 22.2, p < 0.001) differed significantly between groups. Young and O-LP differed, besides age (F(1, 28) = 1827.6, p < 0.001), in MDT (F(1, 28) = 18.7, p = 0.0019), but not in DemTect (F(1, 28) = 4.7, p = 0.1401). Importantly, neither of the measurements revealed pathological results in the older participants. All comparisons were corrected for multiple comparison. The statistical results for the assessment data are provided in the Supp. Material in Tables S2 and S3 as additional information. Effect strength (Cohen’s d) and statistical power are provided in the Supp. Material in Tables S4a and S4b.

### TBSS

#### Young vs O-HP and Young vs O-LP

**Figure 2:**
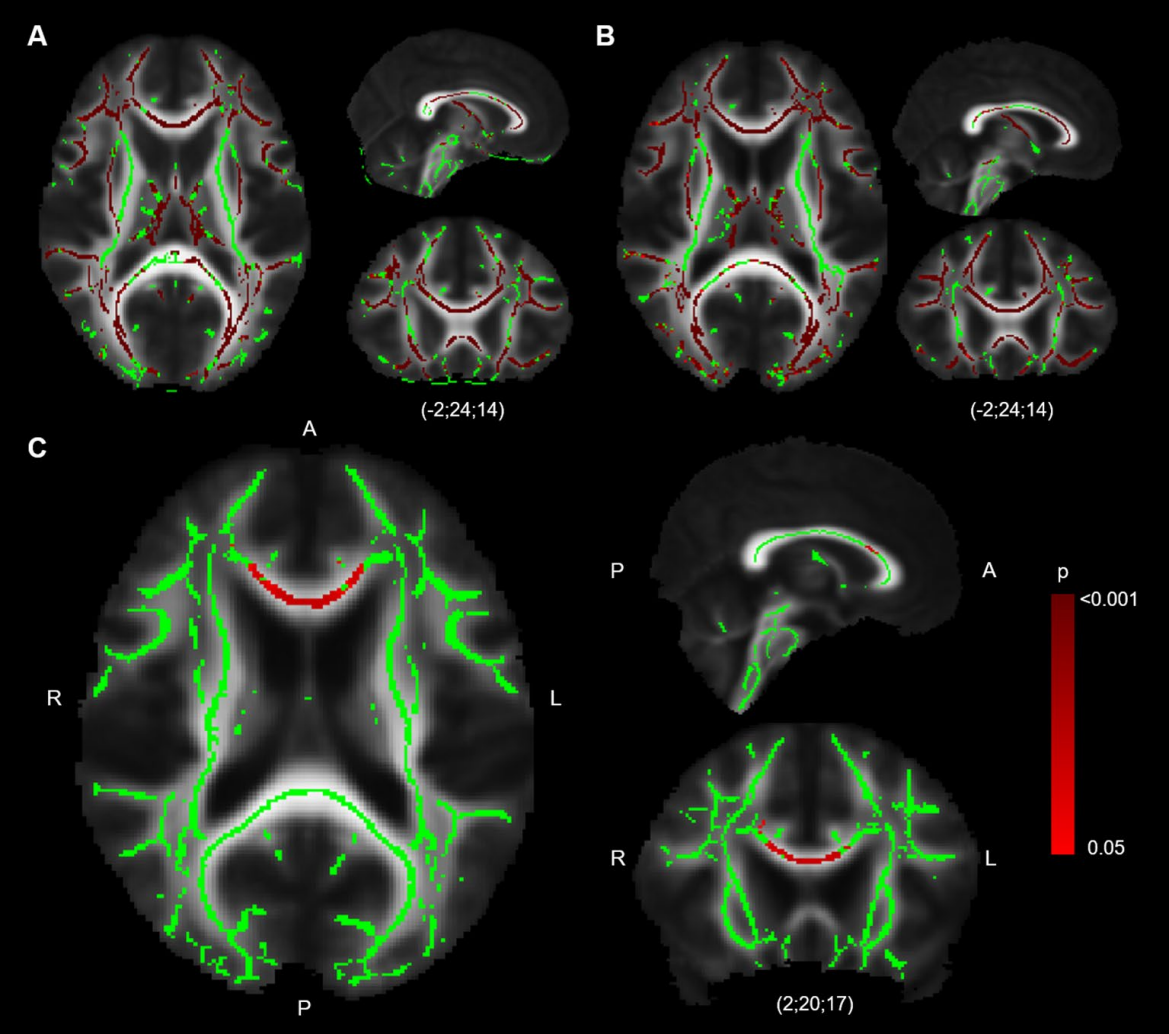
TBSS-Results, FA. TBSS-Results (red), projected on mean-FA-skeleton (green) and mean-FA image. TBSS-Results are thresholded: p < 0.05, FEW-corrected. (**A**) Y > O-HP, (**B**) Y > O-LP, (**C**) O-HP > O-LP, the images are in radiological orientation, coordinates are MNI-coordinates.

Whole brain TBSS-analysis showed significantly higher FA-values for almost every region within the FA-skeleton for the younger participants compared with O-HP and O-LP (see Fig. 2A,B). No region showed higher FA-values for O-HP or O-LP compared with Young.

#### O-HP vs. O-LP

**Figure 3:**
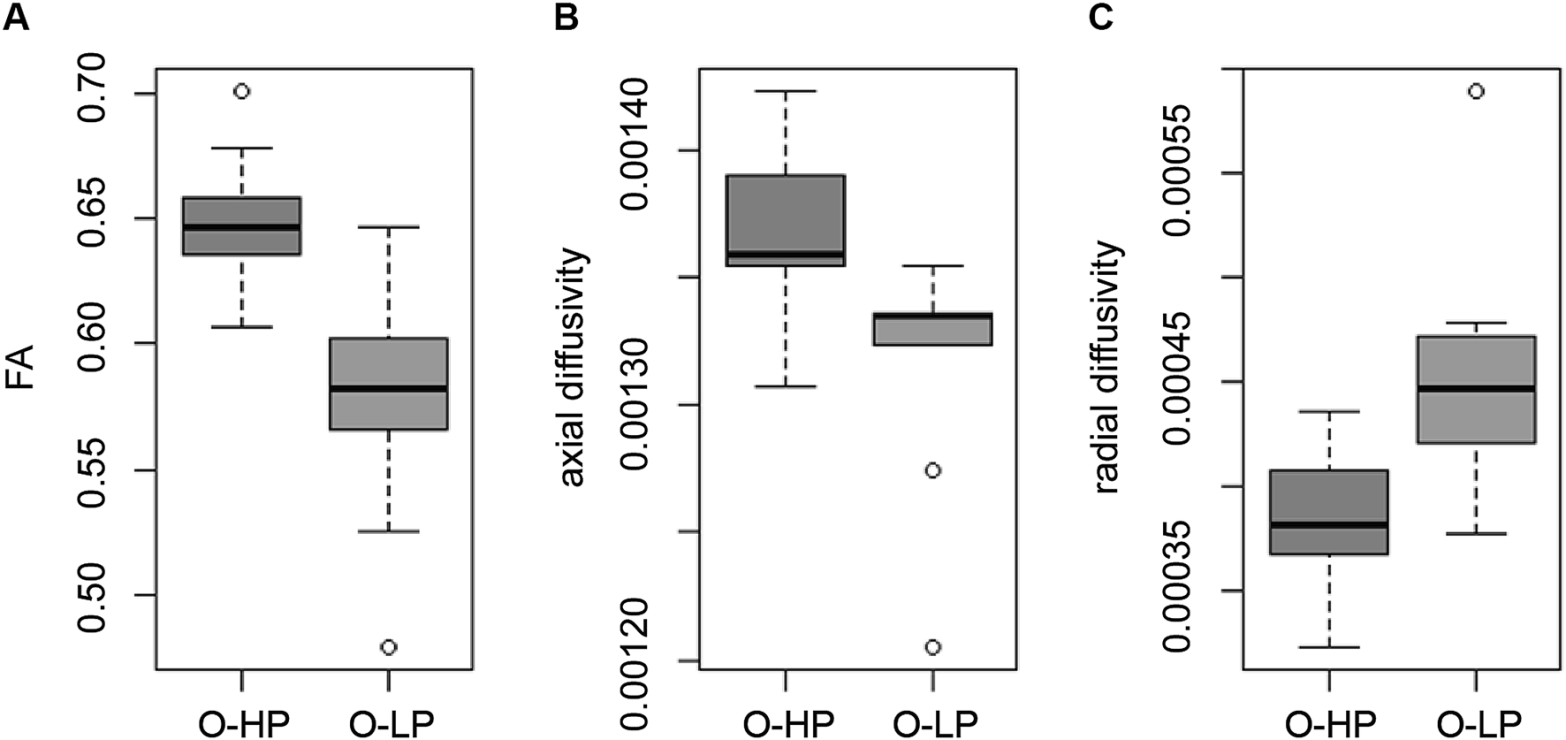
Mean FA, AD and RD for O-HP and O-LP in the anterior corpus callosum. (**A**) mean FA, two-sided t-test, p < 0.001 (O-HP > O-LP), (**B**) mean AD, two-sided t-test, p = 0.001 (O-HP > O-LP), (**C**) mean RD, two-sided t-test, p < 0.001 (O-LP > O-HP).

The whole brain TBSS-analysis with testing for differences between the O-HP and O-LP showed significantly higher FA-values for O-HP, mainly in a region in the anterior part of the corpus callosum and a small region in the right anterior white matter connected to the corpus callosum. This part is equivalent to the overlap between genu and body of the corpus callosum (see Fig. 2C). The mean FA-value of this anterior region showed a significant difference between O-HP (0.65 ± 0.02) and O-LP (0.58 ± 0.05, p < 0.001; see Fig. 3A).

The whole brain TBSS-analysis for AD and RD did not show reliable significant differences between both older groups. To further explore the underlying microstructural alterations in the anterior corpus callosum as defined by the TBSS-FA, we opted to investigate this region in detail by adding a ROI analysis of AD and RD corresponding to the FA approach. As illustrated by Fig. 3B,C, analysis of AD showed significantly lower values for O-LP (0.00132 ± 0.00004) compared to O-HP (0.00137 ± 0.00003, p = 0.001), whereas RD was significantly higher in the group of O-LP (0.00045 ± 0.00006) compared to O-HP (0.00038 ± 0.00003, p < 0.001). Effect strength (Cohen’s d) and statistical power are provided in the Supp. Material in Table S4c as additional information.

### Probabilistic tractography

To further explore the relevance of the TBSS results and to relate our results to the existing literature on the connectivity of the corpus callosum, we used probabilistic tractography to reconstruct the tracts originating from the region in the anterior corpus callosum. This was done for each participant individually with tracts starting from the defined seed mask in the anterior corpus callosum.

**Figure 4:**
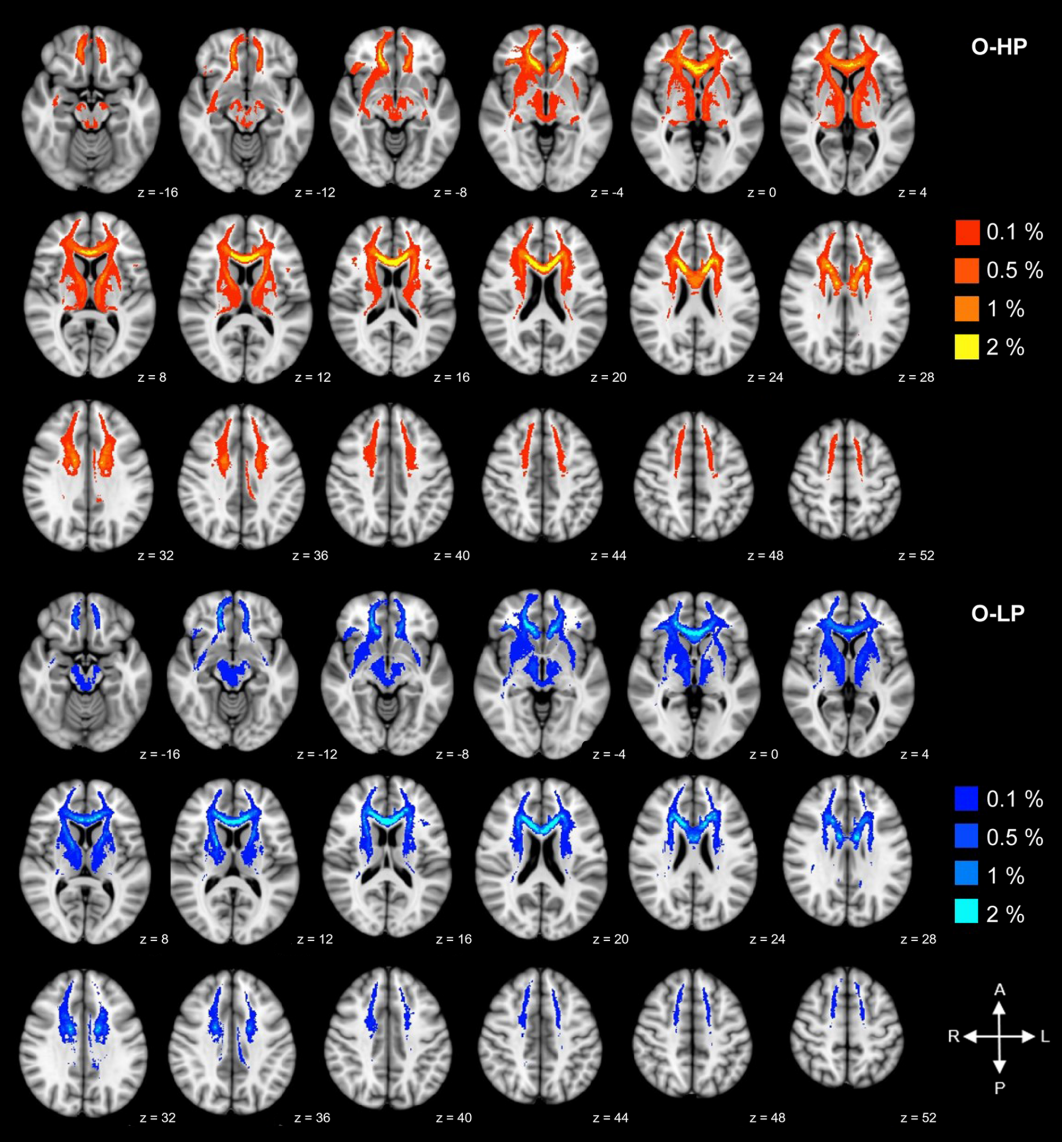
Trajectory map for the resulting tracts for O-HP and O-LP. Superimposed connections resulting from probabilistic tractography on an MNI T1 template for both groups with given z-coordinates. Overlay of binarized group average tracts, thresholded by 50% for both groups. Individual tractography was conducted applying 5000 streamlines per voxel and thresholded by 0.1–2.0% of successful streamlines. The images are all in radiological orientation.

As indicated by Fig. 4, there was a substantial spatial overlay of the trajectory maps for the resulting tracts for O-HP and O-LP. For each threshold, the merged tracts of all participants are plotted, thresholded by 50% of all participants of each group (O-HP and O-LP). The reconstructed tracts showed connections between the seed-ROI and the frontal lobe in both hemispheres, comprising the bilateral frontal pole, the superior frontal, inferior frontal and middle frontal gyrus, areas which are part of the prefrontal cortex. Furthermore, there were relevant connections to subcortical structures such as the bilateral thalamus and the basal ganglia. Visual inspection and statistical comparison did not show significant differences between the two groups.

## Discussion

This study aimed to explore sensory processing in healthy older and younger participants and to test the hypothesis that the integrity of white matter microstructure has an impact on tactile behavioral performance. The data showed that, overall, older participants performed worse in a tactile pattern recognition task. In detail, a subgroup of the older participants showed particular low performance (O-LP), in contrast to another better performing older subgroup (O-HP). Diffusion-weighted imaging helped to better understand this bimodal distribution. The main finding was a significantly reduced microstructural integrity (FA) of transcallosal fibers, particularly in the anterior corpus callosum, in O-LP compared to O-HP. Our data suggest decreased FA in the anterior corpus callosum as a marker of progressed network degradation with aging leading to differences in central processing and, ultimately, performance. The location of the decrease in integrity in frontal brain networks might constitute an explanation for the correlation of measures of sensory acuity with cognitive abilities in older adults.

Multiple previous studies have reported a decrease of FA with increasing age^38,40,43^. During aging, FA in genu and body of the corpus callosum, has been shown to decrease earlier than in other regions (e.g. splenium corpus callosi)^35,62–66^. In the present data, O-HP and O-LP only differed in the FA in the anterior corpus callosum. Alternative diffusion metrics, that were reduced AD and increased RD in the seed region in the anterior corpus callosum, indicated that the reduction of white matter integrity of transcallosal fibers could be a result of age-dependent alterations in both myelinization and axonal integrity^67,68^.

The interpretation of the reduced FA in the anterior corpus callosum as an early marker of structural network degeneration can be supported by its neuroanatomical properties. The anterior part of the corpus callosum mainly contains thinly myelinated, densely packed fibers that connect prefrontal brain areas^40^. These fibers maturate later and exhibit earlier deterioration during aging than the more thickly myelinated fibers in the body and splenium of the corpus callosum, latter connecting motor or sensory areas^40,69,70^. Oligodendrocytes that myelinate the tracts passing the anterior corpus callosum are hypothesized to be among the most metabolically active cells in the adult nervous system. This would make these cells susceptible to the accumulation of metabolic damage and proposes a potential hypothesis on why the anterior corpus callosum might be more vulnerable to aging processes than other brain regions^40,69^.

Taken together, the reduced microstructural integrity found in the present data is consistent with pathophysiological considerations and could be caused by specific properties of the fiber system passing the anterior corpus callosum^44,46,71^.

Regarding the relevance of our findings to sensory processing, the anterior corpus callosum has been shown to mainly connect prefrontal brain areas^40,72,73^. Information flow between distant brain regions is of critical importance for sensory information processing^74^. As discussed in the introduction, sensory processing relies on distributed networks relevant for bottom-up sensory flow and top-down control. Alterations in both might lead to disturbances in tactile recognition. Hence, the identification of specific neuronal networks affected by the decrease in microstructural white matter integrity might give insights into the reasons for poor task performance in O-LP^75^.

We used probabilistic tractography to further relate the local FA reduction in the anterior corpus callosum in O-LP to the underlying structural networks. In line with the literature, probabilistic tractography showed that the seed region in the anterior corpus callosum was connected to the frontal pole, the inferior, middle and superior frontal gyrus, heterogeneous brain regions contributing to prefrontal cortices (PFC), such as the dorsolateral (DFPLC) and ventrolateral (VLPFC) prefrontal cortex and also the orbitofrontal cortex (OFC).

Despite parietal brain regions being the main hub for bottom-up sensory processing, (pre-) frontal brain regions have been shown to be involved in haptic object recognition and body ownership as well^76^. The ventral premotor cortex, for example, contributes to the perceptual awareness of touch and neural activity in the premotor cortex has been linked to the feeling of ownership of the hand^77,78^. Salvini et al. found that even without exploratory movements prefrontal (i.e. the middle, inferior and superior frontal gyrus) and premotor regions are involved in passive tactile recognition of geometrical shapes^79^. Likewise, Hagen et al. found inferior frontal brain regions, namely the posterior inferior frontal gyrus, the adjacent anterior frontal operculum and the OFC to be responsive to somatosensory stimulation^80^. However, both groups could not identify the specificity of the prefrontal activation for somatosensory processing and discuss their findings in the light of higher cognitive functions associated with somatosensory processing, such as working memory, attentional processes and decision making. Accordingly, Hagen et al. speculate that the activation of the VLPFC with tactile stimulation found earlier might also be related to these cognitive components^80^.

In line with this, the PFC has mainly been associated with various higher-order cognitive processes, e.g. working memory^81–83^. More specifically, the DFPLC is known for its role in the executive functions, such as selective attention and cognitive flexibility^84,85^. The VLPFC is an important node in elaborate attentional processes and top-down processing of sensory information^86,87^. The OFC is involved in decision making^88,89^. In addition, the identified region in the anterior corpus callosum was found to be connected bilaterally to the thalamus and the basal ganglia. This is in line with the notion that the cognitive processes mentioned above are also reliant on cortico-subcortical circuits, connecting cortical brain areas with the thalamus^90,91^ and the basal ganglia^92^. We did not find any statistically relevant differences between the probability maps of the two groups. On a speculative note it is possible, that this kind of analysis is not sensitive enough to find small differences between the analyzed groups.

Taken together, probabilistic tractography confirmed that the identified region in the anterior corpus callosum mainly connects prefontal cortices. In line with the literature, we did not detect direct structural pathways between the seed region and parietal brain areas. As elaborated above, disconnection in prefrontal networks could lead to both, disturbances in bottom-up sensory flow and top-down control. However, as there is more evidence for the PFC to be relevant for higher-cognitive functions, we would like to speculate that the reduction of microstructural integrity in the anterior corpus callosum might lead to functional disturbances in frontal cortico-cortical and cortico-subcortical networks relevant for the top-down control of sensory processing. Linking the structural finding to the behavioral results, one could argue that the disturbance of the identified networks might be a possible reason for poor task performance in O-LP, suggesting that poor performance of O-LP seems not to be primarily related to networks relevant for primary central stimulus processing.

To reduce potential confounding factors, we also investigated potential alternative reasons for the group difference between O-HP and O-LP. First, there was no age difference between O-HP and O-LP. Second, there was no difference in peripheral somatosensation between the two groups. In contrast, behavioral tests comparing younger and older individuals pointed to a difference in the MDT, which tended to be worse in the older participants. Of note, this difference became only significant when comparing Young selectively to O-LP. Third, there was no difference in the neuropsychological assessment between O-HP and O-LP. O-LP even had higher scores in DemTect and did not differ significantly from Young. While all participants with pathological test results were excluded, there still was a significant difference between Young and O-HP in DemTect.

Taken together, possible reasons for performance differences between younger and older participants are manifold, comprising differences in peripheral stimuli processing, cognitive decline, but also structural changes of the brain. Comparing O-HP and O-LP, the only difference was found in brain imaging and the regional microstructure in the anterior corpus callosum. We would like to argue that this might be a specific finding showing a progressed aging of the brain and explaining performance differences.

Finally, the location of the FA reduction in networks that might be relevant for both bottom-up sensory processing and top-down control as well as the notion that early sensory decline might be mediated by a deficiency of the prefrontal cortex to exert top-down control during sensory processing establish a link to the correlation between measures of sensory acuity and measures of cognitive abilities in older adults. As both domains rely on frontal brain networks, one could interpret our findings in support of the ‘common cause hypotheses’ of cognitive aging^11,21^.

The found decrease in tactile recognition in O-LP, together with the unremarkable neuropsychological assessment, however, might indicate that sensory processing declines before higher cognitive functions, which would be in favor of the ‘cascade hypothesis’. In this case, one had to ask, why sensory processing would decline before higher cognitive functions, if both are affected by the same underlying neuropathology in the anterior corpus callosum. One explanation, of course, could be, that sensory processing is more vulnerable to the found microstructural alterations. Against that, one could speculate that our tactile pattern recognition task might represent a sensitive marker for a more general early cognitive impairment, potentially more sensitive than the standard neuropsychological measures used in our study. Biological age might not, and the neuropsychological assessment used might not yet reflect the progressed aging of the brain in O-LP. As one of the most essential endeavors in this field is to identify individuals suffering from age-related impairments to allow for early support and interventions, complex tactile recognition might be one asset.

However, due to the found alterations in networks relevant for higher-order cognitive processes, early cognitive impairment might also be assessed by more sensitive measures of higher cognitive functions or complex sensory tasks in other modalities, which rely on the same frontal networks connected by the anterior corpus callosum. A limitation in this sense is that we did not obtain more sensitive measures of higher cognitive functions (like for example the Trail Making Test), which might also have shown group differences. There are some more limitations to the current study. Due to group allocation based on performance, the sample size of O-HP and O-LP differed, which might limit the statistical robustness of the results. Additionally, overall sample sizes were small, and this might question the generalizability of the present results. Especially for any negative results, we cannot preclude that a larger sample size would detect smaller effects. Therefore, our results have to be interpreted carefully. Though, the current study might help to generate hypotheses for further explorations. For instance, prospective studies would help to investigate structural integrity in the anterior corpus callosum over time. Likewise, the effects of behavioral training on FA in this region could be evaluated by future longitudinal studies, which thereby might also infer causality of the present findings. Adding to this, the transfer of our paradigm into fMRI and the evaluation of functional activation patterns in relation to task performance would be a very interesting next step.

In conclusion, we could show an interrelation between microstructural white matter integrity and sensory processing in healthy older adults. The only difference found between older low- and high performers in a tactile pattern recognition task was a decreased microstructural integrity in the anterior corpus callosum. In line with the literature, showing that due to its neuroanatomical properties the anterior corpus callosum is most vulnerable to aging processes, we argue that this might be a specific finding showing an advanced aging of the brain and explaining performance differences. Sensory processing relies on both primary sensory processing and top-down control. As the vulnerable region in the anterior corpus callosum mainly connects prefrontal cortices, our results suggest that disturbances in networks relevant for top-down control of sensory processing are one possible reason for an early decline with aging. The link between impaired tactile recognition and disintegration in frontal brain networks could provide an explanation why the decrease of sensory function correlates with cognitive decline, in line with the ‘common cause hypotheses’ of cognitive aging^11,21^.

## Supplementary Information

Supplementary Information.

## Data Availability

Data will be made available upon request to the corresponding author.
