# Supplementary material for: Reduced frontal white matter microstructure in healthy older adults with low tactile recognition performance
Source: Sci Rep. 2021 Jun 3;11:11689. doi: 10.1038/s41598-021-90995-w (PMC8175740; doi:10.1038/s41598-021-90995-w)

**Supplementary Material**

**Reduced frontal white matter microstructure in healthy older adults with low tactile recognition performance**

*Focko Lorenz Higgen^1^*^#^, Hanna Braaß^1#^, Winifried Backhaus^1^, Robert Schulz^1^, Gui Xue^2^, Christian Gerloff^1^*

^1Department of Neurology, University Medical Center Hamburg-Eppendorf, 20246 Hamburg, Germany^

^2 Key Laboratory of Cognitive Neuroscience and Learning, Beijing Normal University, Beijing, China, 100875^

^#^shared first authorship

^*^Corresponding author:

Focko L. Higgen

Department of Neurology

University Medical Center Hamburg-Eppendorf

Martinistraße 52

20246 Hamburg, Germany

Phone: +49-40-7410-55573

Facsimile: +49-40-7410-57391

Email: f.higgen@uke.de

Table S1a | Mean head displacement for the older participants, calculated using the ecclog-output from eddy correct. All DTI-images were motion corrected with eddy correct (FSL library).

|  | **Mean displacement (ecclog-output) in mm** | | |
| --- | --- | --- | --- |
|  | **x** | **y** | **z** |
| **O-HP** | **0.2 +/- 0.1** | **0.1 +/- 0.04** | **0.44 +/- 0.27** |
| **O-LP** | **0.32 +/- 0.3** | **0.3 +/- 0.5** | **0.67 +/- 0.51** |
| **p (ttest)** | **0.3** | **0.2** | **0.2** |

Table S1b | Mean head displacement for the younger participants, calculated using the ecclog-output from eddy correct.

|  | **Mean displacement (ecclog-output) in mm** | | |
| --- | --- | --- | --- |
|  | **x** | **y** | **z** |
| **Young** | **0.3 +/- 0.2** | **0.2 +/- 0.1** | **0.4 +/- 0.3** |

Table S2 | Statistical results for the assessment data: Assessment related group-differences, lm-results (Y = younger, O-HP = older-high-performers, O-LP = older-low-performers), p-values Benjamini-Yekutieli corrected for multiple comparisons (BY = Benjamini-Yekutieli)

| **Group** | **p (BY-corrected)** |
| --- | --- |
| **O-HP vs O-LP** | 0.65503 |
| **O-HP vs Y** | < 0.0001 |
| **O-LP vs Y** | < 0.0001 |

Table S3 | Statistical results for the assessment data: Assessment related group-differences, MANOVA-results (Y = younger, O-HP = older-high-performers, O-LP = older-low-performers), p-values Benjamini-Yekutieli corrected for multiple comparisons (BY = Benjamini-Yekutieli)

| **p (BY-corr)** | **Y vs O-HP** | **Y vs O-LP** | **O-HP vs O-LP** |
| --- | --- | --- | --- |
| **Age** | <0.0001 | <0.0001 | 0.823 |
| **MDT** | 0.226 | 0.0019 | 1 |
| **2-point-discr.** | 0.767 | 0.078 | 0.915 |
| **MMSE** | 1 | 0.176 | 0.823 |
| **DemTect** | 0.0004 | 0.140 | 0.823 |
| **FEDA-A** | 1 | 0.347 | 0.379 |
| **FEDA-B** | 1 | 0.078 | 0.083 |
| **FEDA-C** | 1 | 0.14 | 0.224 |

Table S4a, S4b and S4c | Cohens’ d and post-hoc power for the assessment data and FA-/RD-/AD-results; post-hoc power was calculated with the R-package “pwr”

| **S4a: Assessment data** | |  |  |  |  |  |
| --- | --- | --- | --- | --- | --- | --- |
| **Cohens**’ **d** | **Y vs O-HP** | **Y vs O-LP** | | | **O-HP vs O-LP** | |
| **Age** | 13.3 | 16.2 | | | 0.52 | |
| **MDT** | 0.77 | 1.5 | | | 0.19 | |
| **2-point-discr.** | 0.32 | 0.9 | | | 0.46 | |
| **MMSE** | 0.33 | 0.74 | | | 0.4 | |
| **DemTect** | 1.51 | 0.87 | | | 0.56 | |
| **FEDA-A** | 0.18 | 0.6 | | | 0.8 | |
| **FEDA-B** | 0.25 | 1 | | | 1.2 | |
| **FEDA-C** | 0.05 | 0.8 | | | 0.95 | |
|  | |  |  |  |  |  |
| **S4b: Assessment data** | |  | | | |  |
| **Post-hoc power** | **Y vs O-HP** | **Y vs O-LP** | | | **O-HP vs O-LP** | |
| **Age** | 1 | 1 | | | 0.25 | |
| **MDT** | 0.65 | 0.96 | | | 0.076 | |
| **2-point-discr.** | 0.16 | 0.61 | | | 0.2 | |
| **MMSE** | 0.17 | 0.45 | | | 0.17 | |
| **DemTect** | 0.996 | 0.58 | | | 0.28 | |
| **FEDA-A** | 0.085 | 0.32 | | | 0.51 | |
| **FEDA-B** | 0.12 | 0.7 | | | 0.84 | |
| **FEDA-C** | 0.05 | 0.51 | | | 0.65 | |
| **S4c: FA-/AD-/RD-results** | | | |  |  |  |

| **O-LP vs O-HP** | **Cohens**’ **d** | **Post-hoc power** |
| --- | --- | --- |
| **FA** | 2.1 | 0.9994 |
| **AD** | 1.49 | 0.96 |
| **RD** | 1.65 | 0.98 |

Figure S1 | Density plots for „accuracy“: Accuracy-plots for each step of the tactile recognition task

*a) Familiarization patterns (800ms)*


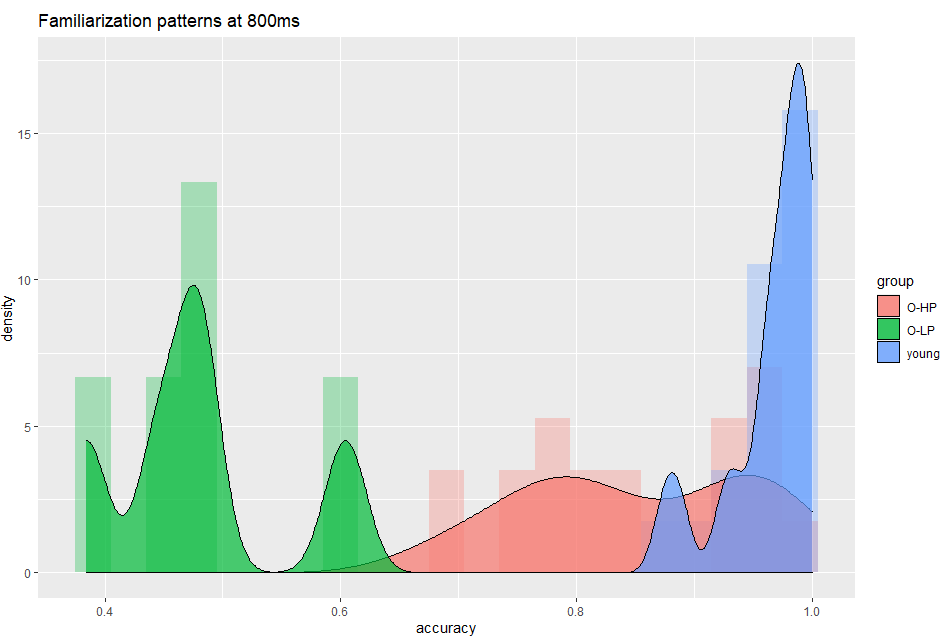


*b) Target patterns at 800ms*


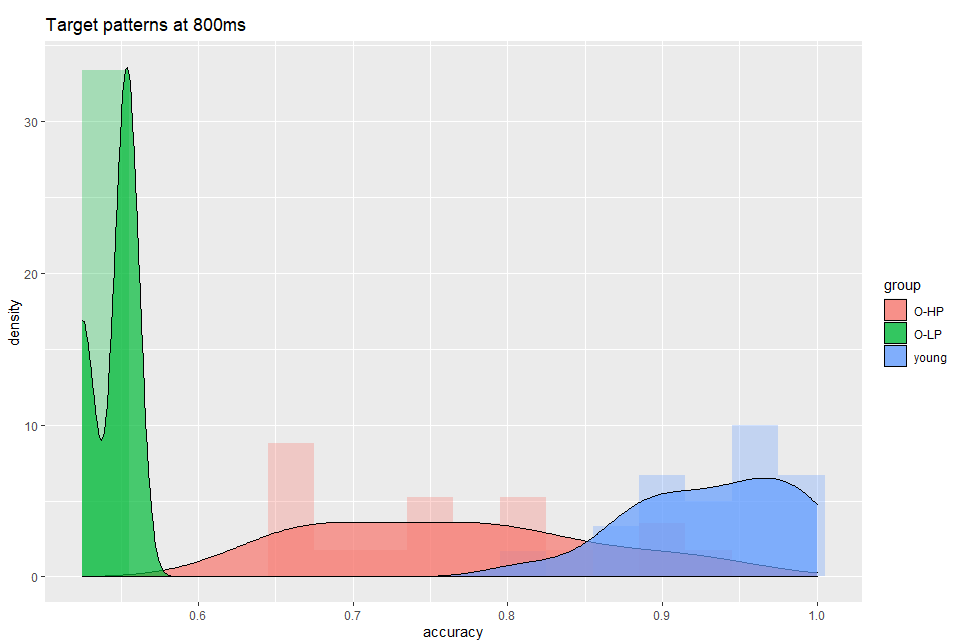


*c) Target patterns at 500ms*


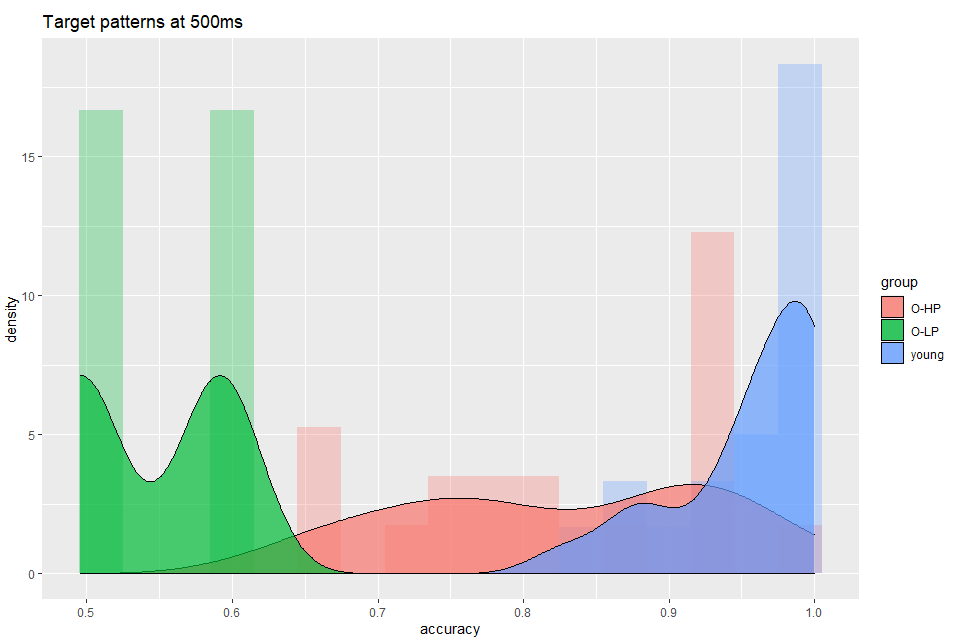

Supplement: Supplementary file 1 — Supplementary Information. [file 41598_2021_90995_MOESM1_ESM.docx]
